# Genome-Wide Association Mapping of Dark Green Color Index using a Diverse Panel of Soybean Accessions

**DOI:** 10.1038/s41598-020-62034-7

**Published:** 2020-03-20

**Authors:** Avjinder S. Kaler, Hussein Abdel-Haleem, Felix B. Fritschi, Jason D. Gillman, Jeffery D. Ray, James R. Smith, Larry C. Purcell

**Affiliations:** 1 Department of Crop, Soil, and Environmental Sciences, University of Arkansas, Fayetteville, AR 72704 USA; 2 USDA-ARS, U.S. Arid Land Agricultural Research Center, 21881 North Cardon Lane, Maricopa, AZ 85138 USA; 3 Division of Plant Sciences, Univ. of Missouri, Columbia, MO 65211 USA; 4 Plant Genetic Research Unit, USDA-ARS, University of Missouri, Columbia, MO 65211 USA; 5 Crop Genetics Research Unit, USDA-ARS, 141 Experimental Station Road, Stoneville, MS 38776 USA

**Keywords:** Natural variation in plants, Agricultural genetics, Plant physiology

## Abstract

Nitrogen (N) plays a key role in plants because it is a major component of RuBisCO and chlorophyll. Hence, N is central to both the dark and light reactions of photosynthesis. Genotypic variation in canopy greenness provides insights into the variation of N and chlorophyll concentration, photosynthesis rates, and N_2_ fixation in legumes. The objective of this study was to identify significant loci associated with the intensity of greenness of the soybean [*Glycine max* (L.) Merr.] canopy as determined by the Dark Green Color Index (DGCI). A panel of 200 maturity group IV accessions was phenotyped for canopy greenness using DGCI in three environments. Association mapping identified 45 SNPs that were significantly (*P* ≤ 0.0003) associated with DGCI in three environments, and 16 significant SNPs associated with DGCI averaged across all environments. These SNPs likely tagged 43 putative loci. Out of these 45 SNPs, eight were present in more than one environment. Among the identified loci, 21 were located in regions previously reported for N traits and ureide concentration. Putative loci that were coincident with previously reported genomic regions may be important resources for pyramiding favorable alleles for improved N and chlorophyll concentrations, photosynthesis rates, and N_2_ fixation in soybean.

## Introduction

Soybean [*Glycine max* (L.) Merr.] is one of the most widely grown crops in the world, and the economic value is primarily derived from the high oil and protein concentrations of the seed. With a protein concentration of around 40%, soybean plants must acquire a large amount of nitrogen (N)^1,2^. In the absence of inorganic N in the soil, symbiotic N_2_ fixation provides N to soybean. Nitrogen fixation reduces N_2_ into biologically useful ammonia (NH_3_) and is carried out by *Bradyrhizobium japonicum* bacteria that live symbiotically in root nodules.

Nitrogen plays a key role in leaf physiology and metabolism because it is a major component of RuBisCO, Photosystems I and II, and chlorophyll; hence, N is central to both the dark and light reaction of photosynthesis^3^. A large amount of N is allocated to the chloroplast (approx. 75%) for synthesis of the photosynthetic apparatus^4^. Leaf N and chlorophyll concentrations are positively correlated across a large range of plant species including maize (Z*ea mays* L.)^5^, rice (*Oryza sativa* L.)^6^, soybean^7,8^, cotton (*Gossypium hirsutum* L.)^9^, and wheat (*Triticum aestivum* L.)^10^. Likewise, there are clear positive relationships between leaf N concentration and photosynthetic rate^7,11–15^. On one hand, a positive correlation between leaf photosynthetic rate and chlorophyll and N concentrations indicates that greener plants would expectantly have higher photosynthesis^12^. On the other hand, reduced chlorophyll concentration can be positively associated with canopy photosynthetic rates^16^ and leaf photosynthetic rates^17^. Recently, Walker *et al*.^18^ used a modelling approach to simulate canopy photosynthesis of genotypes with a range of chlorophyll concentrations, including a chlorophyll-deficient mutant, and found that while canopy photosynthesis may not increase when chlorophyll concentration is reduced, reducing chlorophyll concentration and thus leaf N should be possible while maintaining canopy photosynthetic rates. Variation in canopy greenness among genotypes may provide indirect information on the variation in chlorophyll and N concentrations, leaf photosynthetic rates, and, in legumes, N_2_ fixation. Thus, it may be useful to explore genotypic variation in canopy greenness and associated genetic markers to improvement canopy photosynthesis and/or N_2_ fixation.

A portable chlorophyll meter (such as a SPAD-502, Minolta Corp., Ramsey, NJ) is commonly used to determine leaf greenness and indirectly infer leaf chlorophyll concentration. An alternative method evaluates digital images. In previous research, red, green, and blue (RGB) color components have been used to infer N status of crop plants^19,20^; however, Karcher and Richardson^21^ found that the intensity of red and blue may alter how green an image appears overall. As such, use of a Dark Green Color Index (DGCI) (which is derived from digital values of hue, saturation, and brightness (HSB)) avoids problems from using RGB-derived indices. The DGCI-based measurements of aerial digital images are inexpensive, need little technical expertise, are higher throughput, and allow data acquisition over a much larger area than the small sensor of a SPAD meter.

Understanding the genetic basis of canopy greenness using DGCI could be important for developing cultivars with high N concentration and N_2_ fixation capability and allow increasing the frequency of favorable quantitative trait loci (QTLs) for DGCI alleles. Favorable QTLs can be identified using either genome-wide association mapping or linkage mapping (LM) methods. Major advantages of association mapping over LM include increased mapping resolution, reduced research time, and greater allele number^22^. Advancements in nucleotide sequencing and high-throughput genotyping technologies have facilitated the development of dense molecular-marker datasets, which are almost exclusively composed of single nucleotide polymorphism information (SNPs)^23^. Genotyping diverse lines at thousands of SNPs across the genome is now routine, and permits fine-level genetic mapping through exploiting ancient recombination events^24^. In soybean, 20,087 entries from the USDA germplasm collection (out of 22,500 active accessions, https://npgsweb.ars-grin.gov/gringlobal/taxonomydetail.aspx?id=17711; accessed 12-17-19) have been genotyped using the SoySNP50K iSelect Beadchip (accessible at https://soybase.org/snps/index.php; accessed 12-17-19). This unique soybean genetic resource is proving invaluable for assessing soybean genetic diversity and has opened the door for application of powerful genome wide association mapping methods^25^.

To our knowledge, there has been no report of mapping canopy greenness via DGCI with either bi-parental populations through linkage mapping or association mapping in soybean. However, there are mapping studies of greenness or DGCI in other crop species (including rice^6^ and maize^26^). In soybean, other QTL studies have mapped chlorophyll^27^, N^28^, and ureide concentrations (related to N_2_-fixation)^29^. Our objectives were to use genome wide association mapping to characterize variation of canopy greenness using DGCI in a panel of 200 diverse maturity group (MG) IV accessions, to explore the genetic architecture associated with DGCI, and to predict genotypes with extreme values of DGCI within each MG in the USDA soybean germplasm collection based on the presence of favorable QTLs discovered in the present research.

## Materials and Methods

### Field experiments

The panel of 200 MG IV soybean accessions used for this study consisted of 100 accessions, representing the most genetically diverse accessions (out of 373 accessions) used for previous mapping studies by Kaler *et al*.^30–32^. An additional 100 MG IV accessions were selected from the USDA Soybean Germplasm Collection, based on the estimated breeding values for phenotypes determined from previous association mapping studies^30–32^. These diverse accessions originated from 10 different nations including South Korea, China, Japan, North Korea, Georgia, Russia, Taiwan, India, Mexico, and Romania (Supplementary Table S1). Accessions were evaluated in three environments: the Main Arkansas Agricultural Research Center in Fayetteville, AR (36.15°N, −94.28°) (denoted as “FY”) on a Captina silt loam (Fine-silty, siliceous, active, mesic Typic Fragiudults), the Pine Tree Research Station in Colt, AR (35.12°N, −90.92°) (denoted as “PT”) on a Calloway silt loam (Fine-silty, mixed, active, thermic Aquic Fraglossudalfs), and the Rohwer Research Station in Rohwer, AR (33.80°N, −91.28°) (denoted as “RH”) on a Sharkey silty clay (Very-fine, smectitic, thermic Chromic Epiaquerts). Sowing dates were 7 June 2018 (FY and PT) and 31 May 2018 (RH). Seeds were sown at a density of 37 m^−2^ at a 2.5-cm depth. At FY, plots were 4.57 m long and two rows wide with 0.76 m row spacing. At PT and RH, seeds were sown with a drill (19 cm row spacing), and plots were 1.52 m wide and 4.57 m long. At the PT and RH, the experiment was conducted as an augmented incomplete experimental design with six replications. The FY experiment was conducted with one replication.

### Dark green color index (DGCI) determination

Aerial images were captured using the factory-installed camera (2.54 cm, 20 mega pixel CMOS sensor) of the DJI Phantom 4 Pro (www.dji.com/phantom-4-pro) unmanned aerial system (UAS) which was flown approximately 30.5 m above the ground. The UAS was programmed to collect images with an 80% overlap on the front and sides using Ground Station Pro software from DJI (Shenzhen, China) operating in the ‘3D Map’ mode. The shutter speed was set to ‘auto’ and was programmed to take images at equal time intervals (2 s) with the camera in the nadir position. Image resolution with these settings was approximately 0.8 cm pixel^−1^. Measurements were made 54 (RH), 48 (PT), and 55 (FY) days after sowing when plants were in full bloom and canopies were completely closed. Flights were made between 1100 and 1400 h on days with clear skies. Images were stitched together to form an orthomosaic using Agrisoft Photoscan Professional (www.agrisoft.com). Also included in the image were boards painted with dark green or yellow circles measuring 1 m in diameter. The painted boards had known DGCI values of 0.5722 (green) and 0.0733 (yellow) and served as internal standards for DGCI determination^5,8^. Orthomosaic images were analyzed using FieldAnalyzer software (https://www.turfanalyzer.com/field-analyzer), which was used to extract DGCI values for each plot. Software used the hue (H), saturation (S), and brightness (B) values from a digital image to determine the DGCI value^21^ as shown in the equation below:

$${\rm{DGCI}}\,{\rm{value}}=[({\rm{H}}-60)/60+(1-{\rm{S}})+(1-{\rm{B}})]/3$$

DGCI is a composite number on a scale from 0 to 1 with higher values related to a darker green color and lower values corresponding to a yellow color.

### Statistical analysis of DGCI phenotypes

The PROC UNIVARIATE and PROC CORR procedures, (α = 0.05) of SAS version 9.4 (SAS, Institute 2013) were used for descriptive statistics and Pearson correlation analysis, respectively. We used the PROC MIXED procedure (α = 0.05) of SAS 9.4 for analysis of variance (ANOVA) using a model suggested by Bondari^33^, ${y}_{ijk}=\,\mu +\,{G}_{i}+{E}_{j}+{(GE)}_{ij}+\,{B}_{k(ij)}+\,{\varepsilon }_{ijk},$ where $\mu$ is the total mean, ${G}_{i}$ is the genotypic effect of the ${i}^{th}$ genotype, ${E}_{j}$ is the effect of the ${j}^{th}$ environment, ${(GE)}_{ij}$ is the interaction effect between the ${i}^{th}$ genotype and the ${j}^{th}$ environment, ${B}_{k(ij)}$ is the effect of replication within the ${j}^{th}$ environment, and ${\varepsilon }_{ijk}$ is a random error following $N(0,\,{\sigma }_{e}^{2})$.

Broad sense heritability on an entry-mean basis was estimated using PROC VARCOMP of SAS 9.4 and the Restricted Maximum Likelihood Estimation method. For RH and PT, and across all environments, the Best Linear Unbiased Prediction (BLUP) values were estimated using the PROC MIXED procedure, and BLUP values were used in association mapping analysis. Marker-based narrow sense heritability (*h*^2^) was estimated to understand the variation and trend of predictive ability across traits^34^ using the GAPIT R^35^ package.

### Genotyping and linkage disequilibrium

Single nucleotide polymorphism markers for all 200 accessions were obtained from Soybase (www.soybase.org), providing 42,509 SNPs^25,36^. Genotypic data were cleaned to remove monomorphic markers, and markers with minor allele frequency (MAF) < 5%. Markers with a genotype missing rate >10% were also removed and remaining missing markers datasets were imputed using an LD-kNNi method, which is based on a k-nearest-neighbor-genotype method^37^. A total of 34,680 SNPs were left for association mapping. Linkage disequilibrium (LD) between these markers was measured based on squared correlation coefficients (*r*^2^) of alleles in the TASSEL 5.0 software^38^. A separate LD was calculated for euchromatic and heterochromatic regions. The LD decay with distance was estimated using nonlinear regression, as described by Hill and Weir^39^. The decay rate of LD was determined as the physical distance between markers where the average *r*^2^ dropped to a value of 0.25.

### Genome-wide association analysis

Several statistical models are used for genome wide association mapping. A key consideration for selecting a model is how well it can effectively control false positives that arise from population structure and family relatedness. The Mixed Linear Model (MLM) has often been considered the most popular approach as it considers population structure and family relatedness^22,40^. Since the first publication of MLM for genome wide association mapping^22^, many other MLM-based methods have been developed^40^. These models fail to match the true genetic model of complex traits, which are controlled by many loci simultaneously. Because all of the MLM methods are single-locus and test one marker at a time, they are likely to increase the number of false negatives^41^. To overcome this problem, multi-locus models, such as FASTmrEMMAa and FASTmrMLM^41^, ISIS EM-BLASSO^42^, pLARmEB^43^, pKWmEB^44^, LASSO^45^, and FarmCPU^46^, have been developed. FarmCPU^46^ uses a multi-locus, linear mixed model and iteratively uses fixed and random models with the most significant markers as covariates. This process helps avoid overfitting, reduces the number of reported significant markers and effectively controls for both false positives and false negatives. FarmCPU uses these built-in routines for controlling population structure and family relatedness and has been used successfully in previous soybean association mapping studies^30–32^. In this study, two models, MLM and FarmCPU, were used to compare the DGCI association-mapping results averaged across all environments and to determine which model was more effective in controlling false positives and negatives. Recent research has demonstrated that Bonferroni and other correction methods are too conservative and lead to false negatives when using multi-locus mapping methods^47–49^. Depending upon marker-based heritability^50^, *P-values* of 0.0001^48^, 0.0002^49^, and 0.0003^47^ have been used as appropriate cutoffs in multi-locus association mapping. To consider a SNP significantly associated with DGCI, a threshold value of −Log10 *P* ≥ 3.5 (equivalent to a *P-value* ≤ 0.0003), was used as in previous studies^30–32,51^ and based on the formula developed by Kaler and Purcell^50^. To identify the common significant SNPs present in more than one environment, a threshold value of *P* ≤ 0.05 was allowed but only if the representative SNP had an association of *P* ≤ 0.0003 in at least one additional environment. Using the GAPIT package, we estimated marker based narrow sense heritability using an MLM model as described previously^50^.

### Candidate gene identification and true breeding value determination

Significant SNPs were used to identify candidate genes for DGCI. Genes located within the same LD block that were near SNPs associated with DGCI were considered as potential causative candidate genes. The gene ontologies (GO) associated with candidate genes in the *G. max* genome assembly version Glyma. Wm82.a1.v1.1 and with NCBI RefSeq gene models were obtained from SoyBase (www.soybase.org), and three major GO categories (biological process, cellular component, and molecular function) were assessed. Genes were further classified to be associated with photosynthesis, N metabolic processes and leaf development including aging.

### Allelic effect, favorable alleles, and breeding values estimation

We extrapolated DGCI breeding values for the entire soybean germplasm collection based on calculation of true breeding values as described by Kaler *et al*.^30,32^, which were calculated using the allelic effects and favorable alleles estimated from results of our association-mapping. The difference in mean DGCI between genotypes with the major allele and those with the minor allele was taken as the allelic effect. Alleles were considered as favorable if they were associated with an increase in DGCI, regardless if they were drawn from major or minor allelic classes. SNP effects were expressed as a positive value if the allelic effect increased DGCI. Otherwise, if the allelic effect decreased DGCI then it was expressed as a negative value. All positives and negatives allelic values were summed to estimate the true breeding value of each accession. Based on true breeding values, extreme genotypes were identified from the entire genotyped USDA Soybean Germplasm Collections as having predicted very high or low DGCI values within each MG. the presence of multiple favorable QTLs is associated with a high true breeding value whereas the presence of multiple unfavorable QTLs would be associated with a low true breeding value.

## Results

### Phenotype descriptions

**Figure 1:**
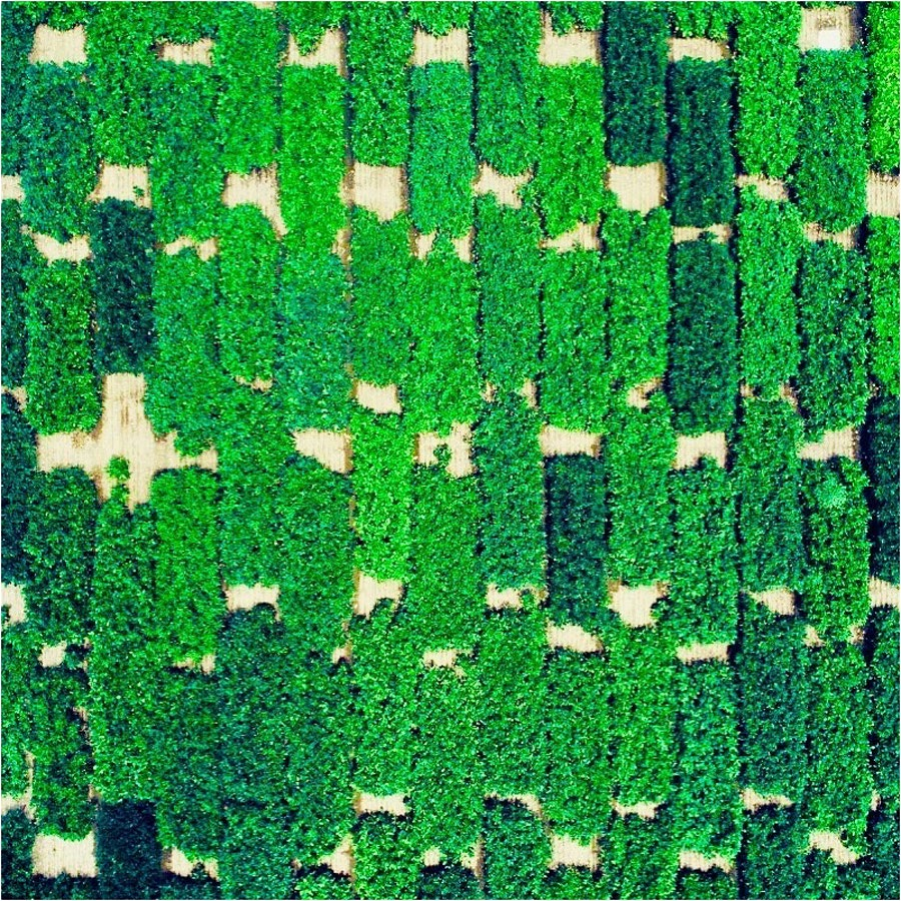
Aerial view of a portion of a field experiment showing large differences in intensity of greenness among soybean accessions.

We observed a broad range of DGCI values within a single environment and when averaged across all environments (Table 1). Visually, there were large differences in the intensity of greenness among accessions (Fig. 1). DGCI had a range of 0.41 (PT), 0.28 (FY), 0.31 (RH), and 0.26 (AVG) (Table 1). The Shapiro–Wilk test of normality was performed, which indicated that DGCI data were normally distributed within each environment and when averaged across all environments (P > 0.01, data not shown); skewness and kurtosis also indicated a normal distribution (Table 1). Analysis of variance of DGCI indicated that there were significant effects for genotype, environment, and genotype by environment interactions (P < 0.05). There were significant positive correlations (P < 0.001) for DGCI between all environments ranging from *r* = 0.46 between PT and FY to *r* = 0.59 between RH and FY (data not shown).

**Table 1:** Broad sense heritability (*H*), marker-based narrow sense heritability (*h*^2^), and descriptive statistics of the dark green color index (DGCI) over 200 MG IV Plant Introductions from experiments conducted at Fayetteville, AR (FY), Pine Tree, AR (PT), Rohwer, AR (RH), and averaged across all environments.

	Pine Tree	Fayetteville	Rohwer	Average
Mean	0.86	0.75	0.73	0.78
Median	0.87	0.76	0.73	0.78
Standard Deviation	0.06	0.05	0.06	0.05
Sample Variance	0.00	0.00	0.00	0.00
Kurtosis	0.95	0.12	−0.32	0.00
Skewness	−0.68	−0.42	0.09	−0.40
Range	0.41	0.28	0.31	0.26
Minimum	0.62	0.59	0.57	0.63
Maximum	1.03	0.87	0.87	0.89
*H*%	59	—	57	75
*h*^2^	44	54	17	37

Broad sense heritability indicates the proportion of phenotypic variation that is explained by genetic effects as a combination of additive effects, dominant/recessive effects, and epistasis. However, marker based narrow sense heritability indicates the proportion of phenotypic variation that is explained by additive genetic effects, and, therefore, is important in plant breeding because the response to selection depends on additive genetic variance. Broad sense heritability for DGCI was moderate to high, ranging from 57% (RH) to 59% (PT) (Table 1). Averaged across all environment, broad sense heritability was 75%. Marker based narrow sense heritability was 44% (PT), 54% (FY), 17% (RH), and 37% when averaged across all environments.

### Genotype data and linkage disequilibrium estimation

A total of 34,680 SNP markers were used for association mapping. These SNPs were more dense in euchromatic regions (an average of 78% of all markers) than heterochromatic regions (an average of 22% of all markers). The SNP distribution in the euchromatic region ranged from 45 SNPs per Mb (Gm19) to 68 SNPs per Mb (Gm09). In the heterochromatic region, SNP distribution ranged from 5 SNPs per Mb (Gm20) to 38 SNPs per Mb (Gm18). LD decayed to *r*^2^ = 0.25 averaged across all chromosomes at 175 kb in the euchromatic region as compared to 5,100 kb in the heterochromatic region. These results were consistent with previous LD decay rates reported for soybean^28,30,52–54^.

### Genome-wide association analysis

**Figure 2:**
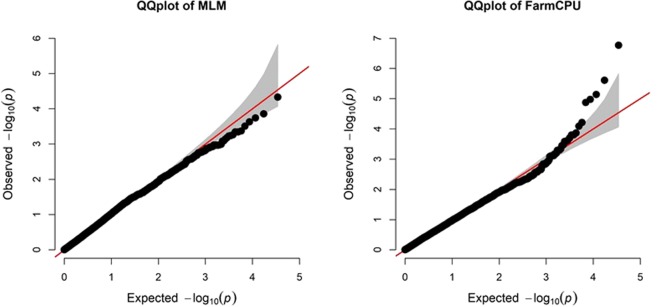
Quantile-quantile (QQ) plot of the mixed linear model (MLM) and FarmCPU model using the dark green color index (DGCI) averaged across all environments.

Average DGCI values across all environments were used to compare the FarmCPU and MLM models (Fig. 2). In the FarmCPU model, the Q-Q plot resulted in a sharp deviation from the expected *P*-value distribution in the tail area, indicating that false positives and negatives were adequately controlled^50^. In contrast, the Q-Q plot for the MLM model did not show a sharp deviation from the expected *P*-value distribution in the tail area (Fig. 2). These results are in agreement with previous results^50^, which collectively demonstrate that the FarmCPU provides better control of type I and type II errors than the MLM model. Therefore, for subsequent association mapping, we only report results for FarmCPU.

**Figure 3:**
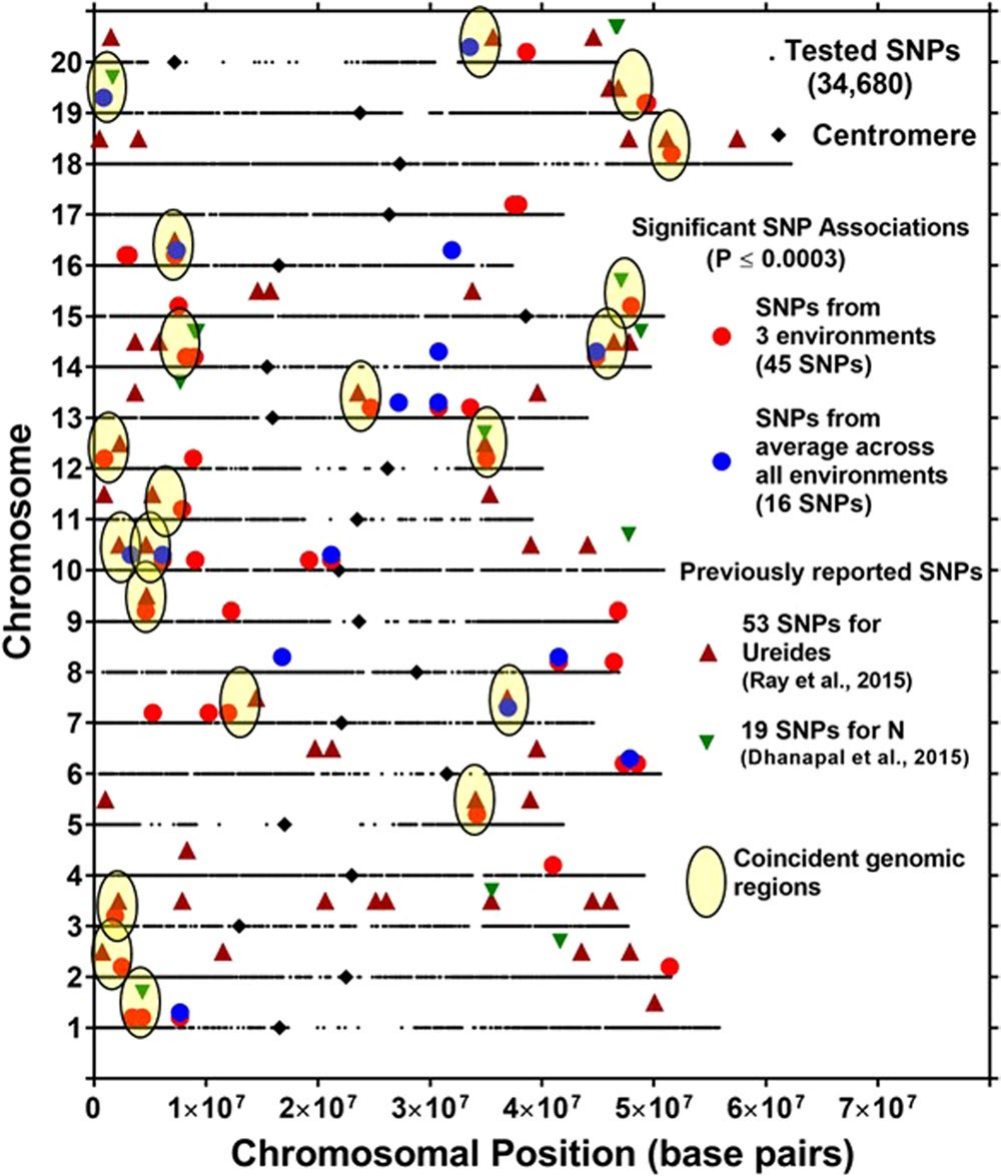
Location of SNPs significantly associated with a dark green color index (DGCI) in three environments and an average across all environments with identified significant SNPs for nitrogen traits^28^ and ureide concentration^29^. *Yellow oval* represents the genomic regions where DGCI was coincident with loci associated with ureides or nitrogen concentration.

Association mapping for DGCI identified 45 significant SNPs in at least one of three environments at a significance level of −Log10 (*P*) ≥ 3.5; *P* ≤ 0.0003 (Fig. 3, Supplementary Fig. S1, and Table 2). Eight out of the 45 SNPs were present in more than one environment. Association mapping identified 16 significant SNPs associated with an averaged DGCI across all environments at a significance level of −Log10 (*P*) ≥ 3.5; *P* ≤ 0.0003 (Fig. 3 and Table 2). Significant SNPs, which were closely spaced and present within the same LD block, were considered as one locus, and out of the 45 significant SNPs from three environments and 16 significant SNPs from the averaged DGCI across all environments, there were 43 putative loci (Table 2, Fig. 3).

**Table 2:** List of significant SNPs associated with dark green color index (DGCI) in three environments, Pine Tree (PT), Rohwer (RH), and Fayetteville (FY), and averaged across all environments (AVG) using the FarmCPU model with the threshold *P* value of (−Log10 (*P*) ≥ 3.5; *P* ≤ 0.0003).

Locus	SNP	CHR	Position	−Log10 (*P*)	Alleles^a^	Allelic effect^b^	%Change^c^	Environment
1	ss715579060	1	3,390,236	3.6	T/C	−0.006	1.5	PT
	ss715579430	1	4,267,470	3.6	A/G	0.003	0.7	PT
2	ss715580803	1	7,659,177	4.8	T/C	0.049	17.5	RH, PT, AVG
3	ss715581591	2	2,458,205	3.7	G/A	0.011	3.9	FY
4	ss715583531	2	51,429,037	3.5	C/T	0.029	10.4	FY, PT
5	ss715584636	3	1,866,786	3.5	C/A	0.029	7.1	PT
6	ss715588053	4	40,982,329	4	C/T	0.002	0.5	PT
	ss715588055	4	40,996,359	3.6	C/A	0.001	0.2	PT
7	ss715591018	5	34,211,795	8.4	T/C	0.071	17.3	PT
8	ss715594787	6	47,315,808	5.6	C/T	0.001	0.4	FY, PT
	ss715594897	6	47,843,257	4.2	G/T	0.003	1.2	AVG
	ss715594979	6	48,475,049	3.5	C/T	−0.03	7.3	PT
9	ss715598313	7	5,226,366	7.1	C/T	0.026	9.3	FY, RH
10	ss715595750	7	10,234,156	4	A/G	0.032	7.8	PT
	ss715595919	7	11,956,773	3.9	T/C	0.051	16.5	RH
11	ss715597487	7	36,972,752	4.1	C/T	0.061	23.5	AVG
12	ss715599860	8	16,790,002	3.8	C/A	0.014	5.4	AVG
13	ss715601931	8	41,504,420	4.3	T/C	0.05	17.9	RH, AVG
14	ss715602501	8	46,430,924	3.7	C/T	0.053	18.9	FY
15	ss715604985	9	4,612,586	4.5	C/T	−0.004	1.4	FY
16	ss715603006	9	12,240,541	4.2	C/A	−0.002	0.5	PT
17	ss715605048	9	46,800,908	3.5	G/A	0.025	8.9	RH
18	ss715606249	10	3,268,393	5.6	T/C	−0.005	1.9	AVG
19	ss715608369	10	6,104,071	4.3	T/C	0.044	15.7	FY, RH, AVG
20	ss715608656	10	9,026,417	5.5	A/G	−0.029	10.4	FY
21	ss715605790	10	19,202,280	3.5	T/G	0.008	2.9	RH
	ss715605845	10	21,174,006	3.7	C/T	0.008	2.9	RH, AVG
22	ss715611154	11	7,846,048	6.7	A/C	0.04	14.3	FY
23	ss715613653	12	896,036	6.1	G/T	−0.003	1.1	FY
24	ss715613628	12	8,844,839	3.7	T/G	−0.019	4.6	PT
25	ss715612526	12	35,036,533	4.2	G/T	−0.006	1.5	PT
26	ss715614254	13	24,708,738	4.3	A/G	0.009	2.2	PT
27	ss715614615	13	27,196,435	3.5	G/A	0.001	0.4	AVG
28	ss715615227	13	30,738,046	3.6	C/A	0.013	5.0	AVG
	ss715615232	13	30,771,524	10.3	A/G	−0.019	6.8	FY
29	ss715615582	13	33,591,479	5.6	T/C	−0.02	4.9	PT
30	ss715619978	14	8,185,171	4.2	A/C	0.035	12.5	FY
	ss715620046	14	8,951,951	5.6	A/G	0.028	10.0	FY
31	ss715618272	14	30,760,829	3.6	C/T	−0.012	4.6	AVG
32	ss715618984	14	44,846,030	4.7	C/T	0.046	16.4	RH
	ss715618985	14	44,854,103	3.5	A/G	0.038	14.6	AVG
33	ss715623028	15	7,522,072	3.5	C/T	−0.015	5.4	FY, PT
34	ss715622385	15	47,961,687	13.5	A/G	0.109	26.6	PT
35	ss715623939	16	2,824,073	4.9	G/T	0.039	13.9	FY, PT,RH
	ss715624366	16	3,067,762	4.6	C/A	0.053	12.9	PT
36	ss715625423	16	7,214,372	4.8	T/C	−0.022	7.9	FY
	ss715625453	16	7,364,708	3.6	G/A	0.02	7.7	AVG
37	ss715624500	16	31,945,745	4.9	G/A	−0.014	5.4	AVG
38	ss715627213	17	37,456,348	3.7	G/A	−0.032	7.8	PT
	ss715627253	17	37,879,524	10.3	A/G	0.021	5.1	PT, RH
39	ss715631221	18	51,574,691	3.7	T/C	−0.019	6.8	FY
40	ss715636405	19	845,338	3.8	G/A	0.059	22.7	AVG
41	ss715635925	19	49,266,400	3.5	A/C	0.012	2.9	PT
	ss715635935	19	49,341,559	3.6	T/C	0.003	0.7	PT
	ss715635938	19	49,388,460	3.7	T/G	0.004	1.0	PT
42	ss715637471	20	33,559,707	5	C/T	−0.037	14.2	AVG
43	ss715638047	20	38,616,560	8.7	C/T	−0.045	11.0	PT

CHR: Glycine max chromosome number.

^a^Allele: Major/Minor alleles of Single Nucleotide Polymorphism.

^b^Allelic effect: Difference in mean DGCI between genotypes with the major allele and those with the minor allele. Positive sign indicates that the major allele is associated with increased DGCI. Negative sign indicates that the minor allele is associated with increased DGCI.

^c^% Change: percentage change in DGCI due to allelic effect.

The allelic effect for the 45 significant loci from three environments and 16 significant loci for an average DGCI across all environments ranged from −0.045 to 0.109 and from −0.037 to 0.061, respectively (Table 2). Eight out of the 45 SNPs, which were present in more than one environment, had allelic effect in the same direction. The percentage change in DGCI value due to the allelic effect was calculated by dividing the absolute value of the allelic effect with the phenotypic range and then multiplying by 100. The percentage change in DGCI associated with a specific allelic effect ranged from 0.2% to 26.6% for three environments and from 0.4% to 23.5% for the average DGCI across all environments. There were 27 SNPs from three environments and 11 SNPs based on the average DGCI across all environments that had a 5% or greater change due to allelic effect.

Allelic effects of all significant loci were used to calculate the true breeding values for DGCI of the entire USDA soybean germplasm collection. Table 3 lists the two accessions from each MG that have the highest and lowest true breeding values for DGCI. These likely represent new genetic sources for improving canopy photosynthesis by optimizing canopy-level light interception in association with leaf N distribution within the canopy. To potentially improve DGCI and N status, a breeding strategy could utilize the information on the favorable alleles with the largest allelic effects (Table 2) with SNP data for specific accessions (https://soybase.org/snps/index.php) to introgress those favorable alleles into elite backgrounds.

**Table 3:** The top two accessions for dark green color index (DGCI) within each maturity group (MG) that have the highest and lowest true breeding values (TBVs), which were summation of all positives and negatives allelic values present in the accession.

	Accession	Province	Country	MG	TBV	Favorable alleles
**Highest**	PI291329	Heilongjiang	China	0	0.907	33
	PI189871	unknown	France	0	0.841	31
	PI189877	unknown	France	00	0.895	35
	PI290155	Pest	Hungary	00	0.895	35
	PI437085	Amur	Russia	000	0.565	28
	PI196501	Ostergotland	Sweden	000	0.557	30
	PI384469A	Krasnodar	Russia	I	0.809	30
	PI437815	Northeast China	China	I	0.789	29
	PI391585	Jilin	China	II	0.845	30
	PI089167	Northeast China	China	II	0.819	30
	PI603912	unknown	North Korea	III	0.899	35
	PI085272	Kyonggi	South Korea	III	0.869	34
	PI458037	Kangwon	South Korea	IV	1.003	34
	PI603397	Liaoning	China	IV	0.987	37
	PI398304	Kyonggi	South Korea	V	0.981	35
	PI509109	Kyongsang Puk	South Korea	V	0.957	35
	PI398332	Kangwon	South Korea	VI	0.925	34
	PI520732	Kyonggi	South Korea	VI	0.925	34
	PI506810	Tohoku	Japan	VII	0.793	30
	PI424475	Cheju	South Korea	VII	0.751	29
	PI200516	Shikoku	Japan	VIII	0.731	28
	PI416819A	Kyushu and Okinawa	Japan	VIII	0.729	30
	PI417084B	Kanto and Tosan	Japan	IX	0.693	30
	PI281894	unknown	Indonesia	IX	0.541	29
	PI240664	Luzon	Philippines	X	0.385	25
	PI567075B	East Java	Indonesia	X	0.337	20
**Lowest**						
	PI603429A	Nei Monggol	China	0	−0.499	17
	PI437257	unknown	Moldova	0	−0.463	12
	PI437528	unknown	Ukraine	00	−0.457	13
	PI437219	unknown	Moldova	00	−0.435	14
	PI507729	Amur	Russia	000	−0.429	15
	PI507823	Amur	Russia	000	−0.429	15
	PI532444A	Jilin	China	I	−0.649	11
	PI461509	Jilin	China	I	−0.599	14
	PI458519A	Jilin	China	II	−0.657	11
	PI464915A	Jilin	China	II	−0.657	11
	PI603550	Shanxi	China	III	−0.907	12
	PI437792	unknown	China	III	−0.889	9
	PI087629	Unknown	Unknown	IV	−0.853	12
	PI548422	Liaoning	China	IV	−0.853	12
	PI548422S	Liaoning	China	V	−0.853	12
	FC031934	unknown	unknown	V	−0.741	12
	PI175194	Uttar Pradesh	India	VI	−0.763	6
	PI578308A	Jumla	Nepal	VI	−0.681	9
	PI165926	Uttar Pradesh	India	VII	−0.681	9
	PI165914	Bihar	India	VII	−0.643	11
	PI323559	Uttar Pradesh	India	VIII	−0.391	14
	PI174854	unknown	Nepal	VIII	−0.385	14
	PI487431	Kagoshima	Japan	IX	−0.399	13
	PI323576	Uttar Pradesh	India	IX	−0.257	15
	PI393551	Hsinchu	Taiwan	X	−0.135	19
	PI518280	Hsinchu	Taiwan	X	−0.135	19

### Candidate gene identification

Genes were considered as potential candidates when they were present within ±175 kb of a significant SNP in euchromatic regions or within ±5,100 kb in heterochromatic regions. These distances represent the distance at which LD decayed to an *r*^2^ = 0.25 in the euchromatic and heterochromatic regions. There were 58 candidate genes associated with DGCI, and these genes are annotated for their gene ontologies (biological process, molecular function, and cellular components) in Supplementary Table S2^54^. Among the interesting annotated biological functions associated with DGCI, there were eight genes annotated for nitrate transport, six genes annotated for chlorophyll, six genes annotated for photosynthesis, six genes annotated for purine transport, six genes annotated for leaf aging and development, three genes annotated for N metabolic processes, and three genes annotated for ammonium metabolism (Supplementary Fig. S2).

## Discussion

The phenotypic variation of canopy greenness using aerial DGCI measurements was determined in a panel of 200 MG IV soybean accessions in three environments. The DGCI varied widely among genotypes, which is important for successful association mapping^24,55^. Significant positive correlations for DGCI between environments and a moderate to high broad sense heritability indicated that DGCI was a relatively stable trait across environments. Marker based narrow sense heritability estimates were moderate to low, which would be expected for a trait, such as DGCI, that is controlled by multiple genes (as indicated in this study) and affected by environment. Low narrow sense heritability estimates indicate that selection for phenotypes in traditional breeding programs would be optimally carried out on pure-lined material and with testing in multiple replications and environments. However, the putative markers identified in this study for DGCI may allow for more rapid progress in breeding than would be expected from traditional approaches.

Similar to the previous studies by Kaler *et al*.^30,31^, the distribution of SNP markers for these 200 accessions varied across genomic regions having fewer gaps in euchromatic regions than in heterochromatic regions. The extent of LD decay in euchromatic and heterochromatic regions was used in this study for gene identification, as was used previously^56^ whereby genes within the same LD block as a QTL were considered as potential candidate genes.

Of the 45 SNPs significantly associated with DGCI in three environments (Fig. 3 and Table 2), 30 major alleles were linked with an increase in DGCI value (Table 2). One locus on Gm15 that had the largest positive allelic effect (0.109) was close to *Glyma15g40911*, which encodes a protein for 2-oxoglutarate and Fe (II)-dependent oxygenase that has a biological function associated with nitrate transport (Supplementary Table S2). Another locus on Gm05 that had the second largest positive allelic effect (0.071) was present close to a gene, *Glyma05g27840*, which codes for a urease annotated as involved with N compound metabolic processes (Supplementary Table S2). A total of 15 minor allele loci identified were associated with an increase in DGCI (Table 2). Of those, one locus on Gm20, with the largest negative allelic effect (−0.045), was present within the coding region of *Glyma20g29850*, which codes an oxalate-CoA ligase annotated as involved with nitrate transport (Supplementary Table S2).

Of the16 SNPs significantly associated with DGCI averaged across all environments, 12 major alleles and four minor alleles were associated with increased DGCI. A major allele on Gm07 that had the largest positive allelic effect (0.061) was located close to *Glyma07g32010*, which codes a MAC/Perforin domain-containing protein with a biological function involved with ammonium transport (Supplementary Table S2). A minor allele on Gm20 that had the largest negative allelic effect (−0.037) was located close to a gene *Glyma20g23750*, which codes a transmembrane transporter annotated as involved in purine nucleobase transport (Supplementary Table S2). Based on the biological functions of these genes, these identified genomic regions and genes are likely determinants of canopy greenness in soybean, and the associated accessions identified in this study with high DGCI may be important resources for incorporating these favorable alleles into new soybean cultivars.

This is the first study identifying QTLs for canopy greenness or DGCI in soybean and complements association mapping studies of chlorophyll traits^57^, N traits^28^, and ureide concentration^29^ in soybean. Loci identified as associated with DGCI in this study were compared with previously reported genomic regions associated with N traits and ureide concentration. We found 21 chromosomal regions that coincide with previously reported genomic regions on Gm01 (1), Gm02 (1), Gm03 (1), Gm05 (1), Gm07 (2), Gm09 (1), Gm10 (2), Gm11 (1), Gm12 (2), Gm13 (1), Gm14 (2), Gm15 (1), Gm16 (1), Gm18 (1), Gm19 (2), and Gm20 (1) (Fig. 3). Interestingly, locus 33 on Gm15 (Table 2), which had the largest allelic effect (0.109) and percent change in DGCI value (26.6%) due to allelic effect, also was associated with chlorophyll a/b ratio^57^ and was coincident with genomic regions identified for N traits^28^ and ureide concentration^29^. These genomic regions had genes with annotated biological functions associated with nitrate (loci 1, 3, 10, 24, 27, 34, 36, 43) or ammonium transport (locus 11), photosystems (loci 9, 12, 21, 37, 38, 40) or response to light (loci 6, 13, 22, 28, 35, 36), leaf senescence (loci 5, 10, 20, 23), chlorophyll biosynthetic processes (loci 27, 30, 33, 36, 39), stomatal complex morphogenesis (loci 32, 41), and purine transport (loci 17, 21, 28, 30, 42) (Supplementary Table S2). These coincident genomic regions for DGCI, ureide concentrations, and N traits may indicate the stability and importance of these loci for canopy chlorophyll and N characteristics. These regions of the genome warrant further investigation, particularly as related to optimizing canopy-level light interception and leaf N distribution to enhance canopy photosynthesis and N use efficiency.

All of our aerial DGCI measurements were collected at full bloom. We have not made comparative measurements of DGCI among genotypes in earlier vegetative stages, but this could potentially provide important information regarding early-season nitrogen acquisition through either nitrogen fixation (on soils with low organic matter and mineralized N) or nitrogen fixation (in soils with low amounts of available N). During seedfill, aerial DGCI measurements in soybean decline^8^. The decrease in DGCI values is accelerated in response to drought. Utilization of aerial DGCI measurements may provide a high throughput method of identifying soybean maturity and of characterizing a shortening of the seed fill period in response to drought^8^.

## Conclusions

This was the first study to map soybean canopy greenness using aerial DGCI measurements. Moderate to high broad sense heritability indicated that DGCI was a relatively stable trait across environments and can be used in soybean breeding programs. We found 45 significant SNPs associated with DGCI in three environments and 16 significant SNPs associated with DGCI averaged across environments. These SNPs likely tagged 43 putative loci. We confirmed 21 chromosomal regions associated with DGCI that were coincident with previously reported genomic regions for chlorophyll a/b ratio, N traits, and ureide concentration. We found 58 candidate genes and 38 of these genes had biological functions associated with nitrate transport, chlorophyll, photosynthesis, purine transport, leaf aging and development, N metabolic process, and ammonium transport. Significant loci that were coincident with previously reported genomic regions, and significant loci that were present in more than one environment, may be an important resource for pyramiding favorable alleles to improve N concentration, leaf and/or canopy photosynthesis rates, and N_2_ fixation ability in soybean breeding programs.

## Supplementary information

Supplementary information.
