# Supplementary material for: Genome-Wide Association Mapping of Dark Green Color Index using a Diverse Panel of Soybean Accessions
Source: Sci Rep. 2020 Mar 20;10:5166. doi: 10.1038/s41598-020-62034-7 (PMC7083947; doi:10.1038/s41598-020-62034-7)
Supplement: Supplementary file 1 — Supplementary information. [file 41598_2020_62034_MOESM1_ESM.docx]

**Supplementary Files**

**Genome-Wide Association Mapping of Dark Green Color Index using a Diverse Panel of Soybean Accessions**

Avjinder S. Kaler^1^, Hussein Abdel-Haleem^2^, Felix B. Fritschi^3^, Jason D. Gillman^4^, Jeffery D. Ray^5^, James R. Smith^5^, and Larry C. Purcell^1*^

^1^Department of Crop, Soil, and Environmental Sciences, University of Arkansas, Fayetteville,

AR 72704, USA

^2^USDA-ARS, U.S. Arid Land Agricultural Research Center, 21881 North Cardon Lane, Maricopa, AZ 85138, USA

^3^Division of Plant Sciences, Univ. of Missouri, Columbia, MO 65211, USA

^4^Plant Genetic Research Unit, USDA-ARS, University of Missouri, Columbia, MO 65211, USA

^5^Crop Genetics Research Unit, USDA-ARS, 141 Experimental Station Road, Stoneville, MS

38776, USA

*corresponding author: [lpurcell@uark.edu](mailto:lpurcell@uark.edu), 1-479-575-3983

**Supplementary** **Figure S1.** Manhattan plots of -Log_10_ (*P*) vs. chromosomal position of significant SNP associations with a dark green color index (DGCI) for three environments; (a) Fayetteville, (b) Pine Tree, (c) Rohwer, and (d) averaged DGCI across all environments using the FarmCPU model. *Black line* represents the association threshold (-Log10 (*P*) ≥ 3.5; *P* ≤ 0.0003). Scale on the right-hand side of each panel indicates the distribution of SNPs across the genome that were used in this study.

**Supplementary Figure S2.** Distribution of biological functions of 38 genes that were directly or indirectly associated with the dark green color index-based measure of leaf greenness.

**Supplementary Table S1.** Maturity group IV soybean accessions along with the country, province, and year of their collection.

**Supplementary Table S2.** List of potential candidate genes associated with dark green color index (DGCI) in soybean and their gene ontologies (GO).

**Supplementary Table S1.** Maturity group IV soybean accessions along with the country, province, and year of their collection.

| Accessions | Province | Country | Year | Maturity GROUP |
| --- | --- | --- | --- | --- |
|  |  |  |  |  |
| FC031630 | unknown | unknown | 1944 | IV |
| PI058955 | Shandong | China | 1924 | IV |
| PI070229 | Jilin | China | 1926 | IV |
| PI080479 | Unknown | Unknown | 1929 | IV |
| PI081030 | Unknown | Unknown | 1929 | IV |
| PI084594 | Unknown | Unknown | 1930 | IV |
| PI084628 | Kyonggi | South Korea | 1930 | IV |
| PI089074 | Unknown | Unknown | 1930 | IV |
| PI090305 | unknown | unknown | 1930 | IV |
| PI092641B | Unknown | Unknown | 1931 | IV |
| PI096280 | Unknown | Unknown | 1932 | IV |
| PI096333 | Hamgyong Puk | North Korea | 1932 | IV |
| PI157396 | unknown | South Korea | 1947 | IV |
| PI157452 | unknown | South Korea | 1947 | IV |
| PI157471 | unknown | South Korea | 1947 | IV |
| PI171433 | Beijing | China | 1948 | IV |
| PI226591 | Tochigi | Japan | 1955 | IV |
| PI229343 | Tohoku | Japan | 1955 | IV |
| PI235335 | unknown | Uruguay | 1956 | IV |
| PI243521 | Akita | Japan | 1957 | IV |
| PI243528 | Akita | Japan | 1957 | IV |
| PI243545 | Akita | Japan | 1957 | IV |
| PI253651A | unknown | China | 1958 | IV |
| PI273483C | Seoul | South Korea | 1961 | IV |
| PI274421 | Miyagi | Japan | 1961 | IV |
| PI360846 | unknown | Japan | 1971 | IV |
| PI361103 | unknown | Korea | 1971 | IV |
| PI377574 | Iwate | Japan | 1973 | IV |
| PI398198 | Seoul | South Korea | 1975 | IV |
| PI398202 | Seoul | South Korea | 1975 | IV |
| PI398223 | Kyonggi | South Korea | 1975 | IV |
| PI398228 | Kyonggi | South Korea | 1975 | IV |
| PI398245 | Kyonggi | South Korea | 1975 | IV |
| PI398249 | Kyonggi | South Korea | 1975 | IV |
| PI398272 | Kyonggi | South Korea | 1975 | IV |
| PI398298 | Kyonggi | South Korea | 1975 | IV |
| PI398321 | Kyonggi | South Korea | 1975 | IV |
| PI398396 | Kangwon | South Korea | 1975 | IV |
| PI398492 | Kangwon | South Korea | 1975 | IV |
| PI398601 | Chungchong Puk | South Korea | 1975 | IV |
| PI398617 | Chungchong Puk | South Korea | 1975 | IV |
| PI398775 | Chungchong Nam | South Korea | 1975 | IV |
| PI398804 | Chungchong Nam | South Korea | 1975 | IV |
| PI398920 | Chungchong Nam | South Korea | 1975 | IV |
| PI398965 | Cholla Nam | South Korea | 1975 | IV |
| PI398970 | Cholla Nam | South Korea | 1975 | IV |
| PI398982 | Kyongsang Puk | South Korea | 1975 | IV |
| PI398987 | Kyongsang Puk | South Korea | 1975 | IV |
| PI398997 | Kyongsang Puk | South Korea | 1975 | IV |
| PI399027 | Kyongsang Nam | South Korea | 1975 | IV |
| PI404164 | unknown | China | 1975 | IV |
| PI404190 | unknown | China | 1975 | IV |
| PI404191 | unknown | China | 1975 | IV |
| PI404199 | unknown | China | 1975 | IV |
| PI407734 | Beijing | China | 1976 | IV |
| PI407735 | Beijing | China | 1976 | IV |
| PI407821A | Kyonggi | South Korea | 1976 | IV |
| PI407892A | Cholla Puk | South Korea | 1976 | IV |
| PI407927B | Cholla Puk | South Korea | 1976 | IV |
| PI408008 | Cholla Nam | South Korea | 1976 | IV |
| PI408021 | Cholla Nam | South Korea | 1976 | IV |
| PI408073 | Kyongsang Puk | South Korea | 1976 | IV |
| PI408100B | Kyongsang Puk | South Korea | 1976 | IV |
| PI408124A | Kyongsang Puk | South Korea | 1976 | IV |
| PI408169D | Kyongsang Puk | South Korea | 1976 | IV |
| PI408173 | Kyongsang Puk | South Korea | 1976 | IV |
| PI408181D | Kyongsang Puk | South Korea | 1976 | IV |
| PI408189 | Kyongsang Puk | South Korea | 1976 | IV |
| PI408209B | Kyongsang Nam | South Korea | 1976 | IV |
| PI408212B | Kyongsang Nam | South Korea | 1976 | IV |
| PI408256 | Kyongsang Nam | South Korea | 1976 | IV |
| PI408262C | Kyongsang Nam | South Korea | 1976 | IV |
| PI408269B | Kyongsang Nam | South Korea | 1976 | IV |
| PI408280 | Kyongsang Nam | South Korea | 1976 | IV |
| PI416858 | Tohoku | Japan | 1977 | IV |
| PI416884 | Tohoku | Japan | 1977 | IV |
| PI416937 | Kanto and Tosan | Japan | 1977 | IV |
| PI416997 | Kanto and Tosan | Japan | 1977 | IV |
| PI417001 | Kanto and Tosan | Japan | 1977 | IV |
| PI417028 | Kanto and Tosan | Japan | 1977 | IV |
| PI417035 | Tohoku | Japan | 1977 | IV |
| PI417070 | Hokuriku | Japan | 1977 | IV |
| PI417276 | Tohoku | Japan | 1977 | IV |
| PI417414B | Kanto and Tosan | Japan | 1977 | IV |
| PI417417 | Kanto and Tosan | Japan | 1977 | IV |
| PI417424 | Kanto and Tosan | Japan | 1977 | IV |
| PI417432 | Tohoku | Japan | 1977 | IV |
| PI417495 | Tohoku | Japan | 1977 | IV |
| PI423741 | Inchon | South Korea | 1978 | IV |
| PI423747B | Kangwon | South Korea | 1978 | IV |
| PI423748C | Kangwon | South Korea | 1978 | IV |
| PI423799A | Chungchong Nam | South Korea | 1978 | IV |
| PI423802 | Chungchong Puk | South Korea | 1978 | IV |
| PI423845A | Chungchong Puk | South Korea | 1978 | IV |
| PI423890B | Akita | Japan | 1978 | IV |
| PI423890C | Akita | Japan | 1978 | IV |
| PI424154A | Kyongsang Puk | South Korea | 1978 | IV |
| PI424231 | Kyonggi | South Korea | 1978 | IV |
| PI424247B | Kangwon | South Korea | 1978 | IV |
| PI424355 | Chungchong Nam | South Korea | 1978 | IV |
| PI424397 | Cholla Puk | South Korea | 1978 | IV |
| PI424399 | Cholla Puk | South Korea | 1978 | IV |
| PI424401 | Cholla Puk | South Korea | 1978 | IV |
| PI424435 | Cholla Nam | South Korea | 1978 | IV |
| PI424488B | Kyongsang Nam | South Korea | 1978 | IV |
| PI424513 | Kyongsang Nam | South Korea | 1978 | IV |
| PI424546A | Kyongsang Puk | South Korea | 1978 | IV |
| PI424614 | Kyongsang Puk | South Korea | 1978 | IV |
| PI430598A | Fujian | China | 1978 | IV |
| PI432359 | Jalisco | Mexico | 1979 | IV |
| PI437745 | unknown | China | 1980 | IV |
| PI442006 | Kyonggi | South Korea | 1980 | IV |
| PI442012A | Kyonggi | South Korea | 1980 | IV |
| PI458084 | Kangwon | South Korea | 1981 | IV |
| PI458098 | Kangwon | South Korea | 1981 | IV |
| PI458119 | Chungchong Nam | South Korea | 1981 | IV |
| PI458515 | Shandong | China | 1981 | IV |
| PI468923 | Beijing | China | 1982 | IV |
| PI471938 | unknown | Nepal | 1982 | IV |
| PI476939 | (north) | Vietnam | 1983 | IV |
| PI495017B | Beijing | China | 1985 | IV |
| PI506519 | Tohoku | Japan | 1986 | IV |
| PI506937 | Tohoku | Japan | 1986 | IV |
| PI507025 | Hokkaido | Japan | 1986 | IV |
| PI507067 | Kanto | Japan | 1986 | IV |
| PI507073 | Tohoku | Japan | 1986 | IV |
| PI507311 | Kanto | Japan | 1986 | IV |
| PI507395 | Kanto | Japan | 1986 | IV |
| PI507407 | Kanto | Japan | 1986 | IV |
| PI507408 | Kanto | Japan | 1986 | IV |
| PI507424 | Kanto | Japan | 1986 | IV |
| PI507435 | Kanto | Japan | 1986 | IV |
| PI507446 | Kanto | Japan | 1986 | IV |
| PI507449 | Kanto | Japan | 1986 | IV |
| PI509079 | Chungchong Nam | South Korea | 1987 | IV |
| PI518673 | Illinois | United States | 1981 | IV |
| PI532462B | Hebei | China | 1989 | IV |
| PI532466A | Chungchong Puk | South Korea | 1989 | IV |
| PI538380 | Hebei | China | 1989 | IV |
| PI548169 | Illinois | United States | 1954 | IV |
| PI548250 | Iowa | United States | 1982 | IV |
| PI548314 | Illinois | United States | 1940 | IV |
| PI548359 | Beijing | China | 1931 | IV |
| PI548427 | Liaoning | China | 1909 | IV |
| PI548430 | Liaoning | China | 1918 | IV |
| PI548431 | Liaoning | China | 1966 | IV |
| PI561303 | unknown | China | 1991 | IV |
| PI567201D | unknown | Georgia | 1992 | IV |
| PI567202 | unknown | Georgia | 1992 | IV |
| PI567272B | unknown | Taiwan | 1992 | IV |
| PI567291 | Gansu | China | 1992 | IV |
| PI567447A | Shanxi | China | 1992 | IV |
| PI567482B | Hebei | China | 1992 | IV |
| PI567488A | Hebei | China | 1992 | IV |
| PI567488B | Hebei | China | 1992 | IV |
| PI567488C | Hebei | China | 1992 | IV |
| PI567489B | Hebei | China | 1992 | IV |
| PI567491B | Hebei | China | 1992 | IV |
| PI567527 | Shandong | China | 1992 | IV |
| PI567531 | Shandong | China | 1992 | IV |
| PI567532 | Shandong | China | 1992 | IV |
| PI567548 | Shandong | China | 1992 | IV |
| PI567572B | Shandong | China | 1992 | IV |
| PI567593B | Shandong | China | 1992 | IV |
| PI567616 | Henan | China | 1992 | IV |
| PI567659 | Henan | China | 1992 | IV |
| PI567753C | Jiangsu | China | 1992 | IV |
| PI567762A | Jiangsu | China | 1992 | IV |
| PI567777 | Jiangsu | China | 1992 | IV |
| PI574477 | Shanxi | China | 1992 | IV |
| PI578494A | Shanxi | China | 1994 | IV |
| PI587588A | Jiangsu | China | 1994 | IV |
| PI594160 | Akita | Japan | 1996 | IV |
| PI594289 | Akita | Japan | 1996 | IV |
| PI594399C | Anhui | China | 1996 | IV |
| PI594410 | Anhui | China | 1996 | IV |
| PI594664 | Guizhou | China | 1996 | IV |
| PI597480A | unknown | South Korea | 1997 | IV |
| PI597485 | unknown | South Korea | 1997 | IV |
| PI603166 | unknown | North Korea | 1997 | IV |
| PI603171 | unknown | North Korea | 1997 | IV |
| PI603418D | Liaoning | China | 1998 | IV |
| PI603454 | Shandong | China | 1998 | IV |
| PI603457A | Shandong | China | 1998 | IV |
| PI603458A | Shandong | China | 1998 | IV |
| PI603465C | Shandong | China | 1998 | IV |
| PI603489 | Shandong | China | 1998 | IV |
| PI603490 | Shandong | China | 1998 | IV |
| PI603543B | Shanxi | China | 1998 | IV |
| PI603554B | Shanxi | China | 1998 | IV |
| PI603555 | Shanxi | China | 1998 | IV |
| PI603563B | Shanxi | China | 1998 | IV |
| PI603692 | Jiangsu | China | 1998 | IV |
| PI603909B | unknown | North Korea | 1998 | IV |
| PI603911C | unknown | North Korea | 1998 | IV |
| PI603917 | unknown | North Korea | 1998 | IV |
| PI606435 | (north) | Vietnam | 1998 | IV |
| PI612612A | unknown | North Korea | 1998 | IV |
| PI632428 | Ohio | United States | 2004 | IV |
| PI642055 | Mississippi | United States | 2006 | IV |

**Supplementary Table S2.** List of potential candidate genes associated with dark green color index (DGCI) in soybean and their gene ontologies (GO).

| **Locus** | **SNP** | **Gene** | **Biological Process** | **Molecular Function** | **Cellular Component** |
| --- | --- | --- | --- | --- | --- |
| 1 | ss715579060 | Glyma01g03930 | gluconeogenesis; tRNA aminoacylation for protein translation | aminoacyl-tRNA ligase activity; | mitochondrion; plasma membrane; chloroplast; |
|  | ss715579430 | Glyma01g04830 | nitrate transport | low affinity nitrate transmembrane transporter activity | plasma membrane |
| 2 | ss715580803 | Glyma01g07120 | nucleobase-containing compound transport | uracil:cation symporter activity | plasma membrane |
| 3 | ss715581591 | Glyma02g02680 | nitrate transport | low affinity nitrate transmembrane transporter activity | plasma membrane; membrane |
| 4 | ss715583531 | Glyma02g47890 | N-terminal protein myristoylation | AMP-activated protein kinase activity | cytoplasm |
| 5 | ss715584636 | Glyma03g02090 | regulation of transcription, negative regulation of leaf senescence | sequence-specific DNA binding transcription factor activity | nucleus |
| 6 | ss715588053 | Glyma04g34745 | response to red or far red light | zinc ion binding | nucleus |
|  | ss715588055 | Glyma04g34770 | acetyl-CoA metabolic process; calcium ion transport; divalent metal ion transport | dimethylallyltranstransferase activity | cytoplasm; cytosol |
| 7 | ss715591018 | Glyma05g27840 | nitrogen compound metabolic process; urea metabolic process | urease activity | cytoplasm |
| 8 | ss715594787 | Glyma06g44390 | biological process | molecular function | mitochondrion; peroxisomal membrane; chloroplast |
|  | ss715594897 | Glyma06g45043 | polyamine catabolic process; flavonol biosynthetic process | luteolin O-methyltransferase activity | nucleus; cytoplasm; cytosol; plasma membrane; plasmodesma |
|  | ss715594979 | Glyma06g45740 | vegetative to reproductive phase transition of meristem; carpel development | histone methyltransferase activity (H3-K4 specific) | nucleus |
| 9 | ss715598313 | Glyma07g06550 | photosynthesis | molecular function | mitochondrion; chloroplast |
| 10 | ss715595750 | Glyma07g11810 | nitrate assimilation; leaf senescence | glutamate-ammonia ligase activity; copper ion binding | cell wall; cytoplasm; cytosol; plasma membrane; plasmodesma |
|  | ss715595919 | Glyma07g13380 | microtubule cytoskeleton organization | translation initiation factor activity | extracellular region; cytoplasm |
| 11 | ss715597487 | Glyma07g32010 | anion transport; ammonium transport | MAC/Perforin domain-containing protein | nucleus |
| 12 | ss715599860 | Glyma08g22130 | photosystem II assembly; protein import into chloroplast stroma | NA | chloroplast; chloroplast inner membrane; membrane |
| 13 | ss715601931 | Glyma08g41500 | polysaccharide biosynthetic process; anthocyanin accumulation in tissues in response to UV light | protein serine/threonine kinase activity; | plasma membrane |
| 14 | ss715602501 | Glyma08g41544 | biological process | molecular function | nucleus; chloroplast |
| 15 | ss715604985 | Glyma09g05820 | PSII associated light-harvesting complex II catabolic process | metalloendopeptidase activity; zinc ion binding | mitochondrion; chloroplast; chloroplast thylakoid |
| 16 | ss715603006 | Glyma09g11970 | glycolysis; ubiquitin-dependent protein catabolic process; aerobic respiration; photorespiration | catalytic activity | mitochondrion; mitochondrial respiratory chain complex I; membrane |
| 17 | ss715605048 | Glyma09g42150 | purine nucleobase transport | transporter activity | membrane; integral to membrane |
| 18 | ss715606249 | Glyma10g04300 | organ morphogenesis; tissue developmen | transporter activity; ATP binding | nucleus; chromosome; cohesin complex; chloroplast |
| 19 | ss715608369 | Glyma10g07340 | gluconeogenesis; cellular amino acid metabolic process; threonine metabolic process | L-threonine ammonia-lyase activity; pyridoxal phosphate binding | chloroplast |
| 20 | ss715608656 | Glyma10g09750 | leaf senescence | molecular function | mitochondrion; chloroplast |
| 21 | ss715605790 | Glyma10g16420 | chloroplast organization; photosynthetic electron transport in photosystem II; cytochrome b6f complex assembly | mRNA 3'-UTR binding; translation release factor activity | cytoplasm; chloroplast; chloroplast stroma |
|  | ss715605845 | Glyma10g17590 | purine nucleotide biosynthetic process; leaf development | aminoacyl-tRNA ligase activity | cytoplasm; mitochondrion; chloroplast; chloroplast stroma |
| 22 | ss715611154 | Glyma11g11020 | polysaccharide biosynthetic process; response to light stimulus; anatomical structure morphogenesis; anthocyanin accumulation in tissues in response to UV light | UDP-N-acetylmuramate dehydrogenase activity; flavin adenine dinucleotide binding | nucleus; vacuole; vacuolar membrane; plasma membrane; membrane; integral to membrane |
| 23 | ss715613653 | Glyma12g01250 | protein lipidation; autophagy; aging; leaf senescence; cellular response to phosphate starvation | APG8 activating enzyme activity | cytoplasm; cytosol |
| 24 | ss715613628 | Glyma12g10841 | nitrate transport | peroxidase activity; heme binding | extracellular region; endoplasmic reticulum |
| 25 | ss715612526 | Glyma12g31100 | polyamine biosynthetic process; nitrogen compound metabolic process; | hydrolase activity, acting on carbon-nitrogen (but not peptide) bonds | NA |
| 26 | ss715614254 | Glyma13g21211 | protein peptidyl-prolyl isomerization; peptidyl-proline modification | peptidyl-prolyl cis-trans isomerase activity | nucleus; chloroplast; thylakoid |
| 27 | ss715614615 | Glyma13g23890 | nitrate transport; chlorophyll catabolic process | transcription regulatory region sequence-specific DNA binding; protein homodimerization activity | nucleus |
| 28 | ss715615227 | Glyma13g27650 | purine ribonucleoside monophosphate biosynthetic process | adenosine deaminase activity; deaminase activity | cytoplasm |
|  | ss715615232 | Glyma13g28180 | nitrogen compound metabolic process; response to blue light; response to high light intensity; ammonia assimilation cycle | glutamate-ammonia ligase activity | mitochondrion; chloroplast; chloroplast thylakoid membrane; chloroplast stroma; thylakoid |
| 29 | ss715615582 | Glyma13g31120 | embryo development ending in seed dormancy | NA | plasma membrane |
| 30 | ss715619978 | Glyma14g10040 | purine ribonucleoside salvage; adenosine salvage | copper ion binding; kinase activity | cytoplasm; cytosol; plasma membrane |
|  | ss715620046 | Glyma14g10780 | biological process; chlorophyll catabolic process | molecular function | chloroplast |
| 31 | ss715618272 | Glyma14g25413 | protein phosphorylation; thylakoid membrane organization; vegetative to reproductive phase transition of meristem | protein serine/threonine kinase activity; calcium ion binding | plasma membrane |
| 32 | ss715618984 | Glyma14g35750 | stomatal complex morphogenesis; stamen development | ubiquitin-protein ligase activity | nucleus |
|  | ss715618985 | Glyma14g35770 | gibberellin biosynthetic process; response to abscisic acid stimulus | DNA binding; sequence-specific DNA binding transcription factor activity; protein binding | nucleus |
| 33 | ss715623028 | Glyma15g10440 | leaf morphogenesis; chlorophyll biosynthetic process | 3,8-divinyl protochlorophyllide a 8-vinyl reductase activity | mitochondrion; chloroplast; chloroplast thylakoid; chloroplast envelope; membrane |
| 34 | ss715622385 | Glyma15g40911 | nitrate transport | oxidoreductase activity | cytoplasm |
| 35 | ss715623939 | Glyma16g03320 | response to high light intensity; vegetative to reproductive phase transition of meristem | calmodulin binding; chaperone binding | nucleus; plasmodesma |
|  | ss715624366 | Glyma16g03640 | response to ethylene stimulus; response to auxin stimulus; response to gibberellin stimulus; photoperiodism, flowering | DNA binding; sequence-specific DNA binding transcription factor activity | nucleus |
| 36 | ss715625423 | Glyma16g08040 | response to light stimulus; chlorophyll biosynthetic process; | zinc ion binding; enone reductase activity; 2-alkenal reductase (NADP+) activity | chloroplast; chloroplast stroma; thylakoid; chloroplast envelope; stromule; apoplast |
|  | ss715625453 | Glyma16g08150 | anion transport; detection of mechanical stimulus; nitrate transport | mechanically-gated ion channel activity | nucleus; plasma membrane; membrane |
| 37 | ss715624500 | Glyma16g27995 | photosynthesis, light harvesting in photosystem II; | chlorophyll binding | chloroplast; chloroplast thylakoid; |
| 38 | ss715627213 | Glyma17g33570 | calcium-mediated signaling; leaf development; protein stabilization | protein binding; ATP binding; ATPase activity | cell wall; nucleus; cytoplasm; mitochondrion; Golgi apparatus |
|  | ss715627253 | Glyma17g33950 | pyrimidine ribonucleotide biosynthetic process; photomorphogenesis; regulation of flower development | 3-hydroxyisobutyryl-CoA hydrolase activity | mitochondrion |
| 39 | ss715631221 | Glyma18g42540 | chlorophyll biosynthetic process; cellular response to phosphate starvation | UDP-glycosyltransferase activity | plasma membrane; chloroplast; plastid; chloroplast envelope |
| 40 | ss715636405 | Glyma19g01260 | photosynthetic electron transport in photosystem I; photosystem II stabilization | peptidyl-prolyl cis-trans isomerase activity | chloroplast; chloroplast thylakoid; |
| 41 | ss715635925 | Glyma19g43700 | protein targeting to peroxisome; embryonic morphogenesis | protein C-terminus binding; zinc ion binding | intracellular; peroxisome; peroxisomal membrane |
|  | ss715635935 | Glyma19g43780 | biological process; stomatal complex morphogenesis | NA | mitochondrion; chloroplast |
|  | ss715635938 | Glyma19g43815 | regulation of transcription, DNA-dependent; defense response; ethylene mediated signaling pathway | DNA binding; sequence-specific DNA binding transcription factor activity | nucleus |
| 42 | ss715637471 | Glyma20g23750 | purine nucleobase transport | transmembrane transporter activity | membrane; integral to membrane |
| 43 | ss715638047 | Glyma20g29850 | nitrate transport | AMP binding; oxalate-CoA ligase activity | cytoplasm; mitochondrion; plasmodesma; chloroplast; |


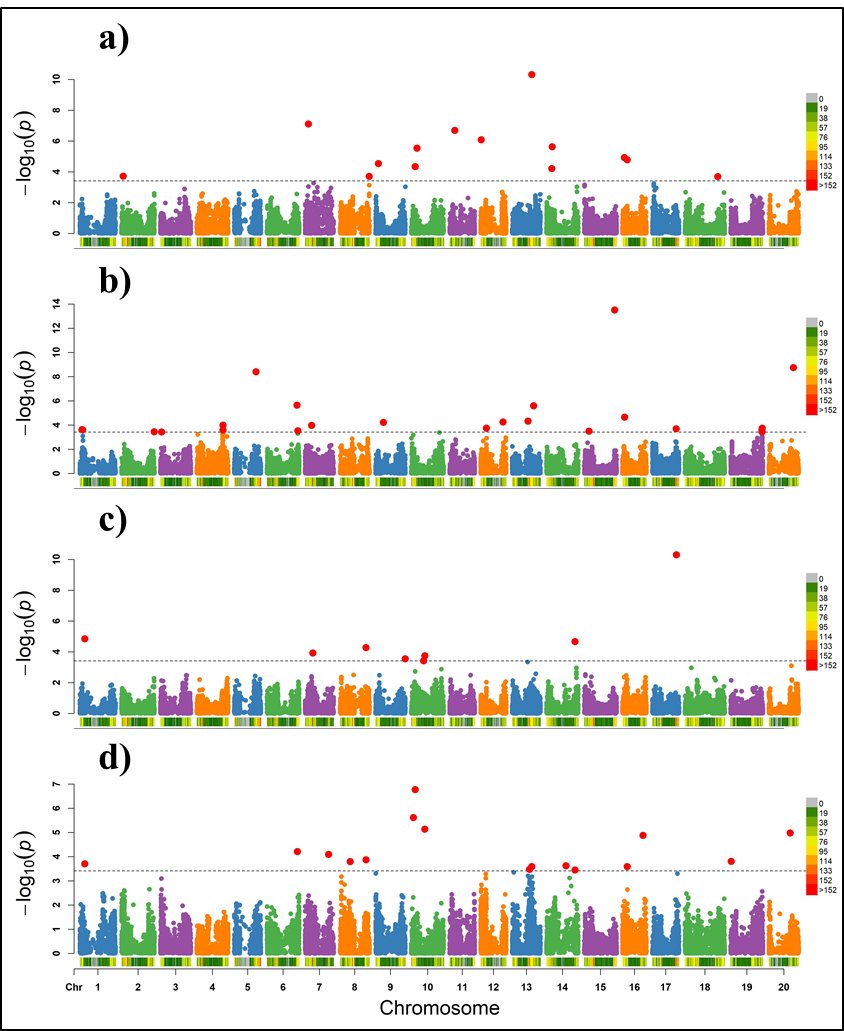


**Figure S1.** Manhattan plots of -Log_10_ (*P*) vs. chromosomal position of significant SNP associations with a dark green color index (DGCI) for three environments; (a) Fayetteville, (b) Pine Tree, (c) Rohwer, and (d) averaged DGCI across all environments using the FarmCPU model. *Black line* represents the association threshold (-Log10 (*P*) ≥ 3.5; *P* ≤ 0.0003). Scale on the right-hand side of each panel indicates the distribution of SNPs across the genome that were used in this study.


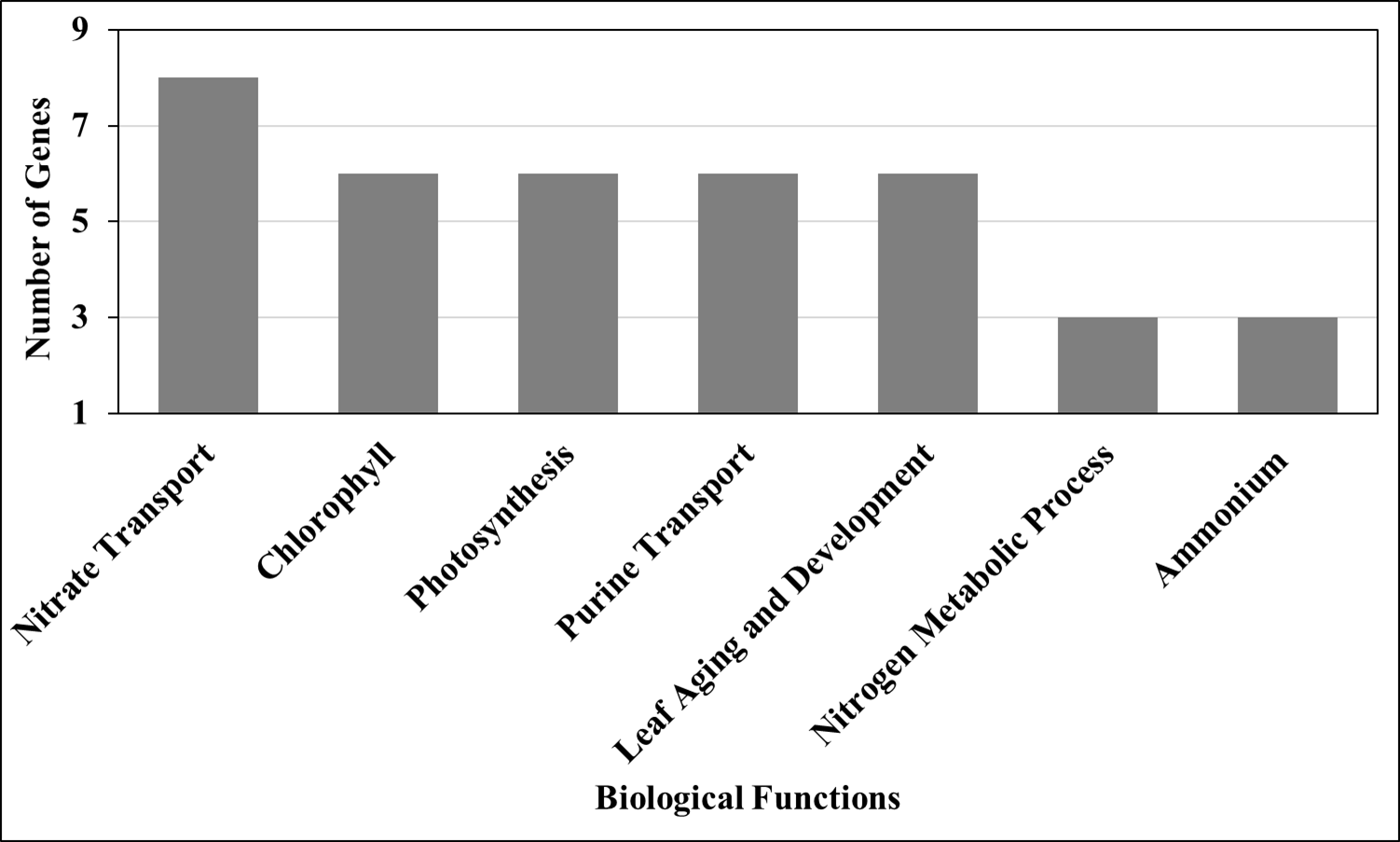


**Figure S2.** Distribution of biological functions of 38 genes that were directly or indirectly associated with the dark green color index-based measure of leaf greenness.
